# Effective Conductivity of Carbon-Nanotube-Filled Systems by Interfacial Conductivity to Optimize Breast Cancer Cell Sensors

**DOI:** 10.3390/nano12142383

**Published:** 2022-07-12

**Authors:** Yasser Zare, Kyong-Yop Rhee, Soo-Jin Park

**Affiliations:** 1 Biomaterials and Tissue Engineering Research Group, Department of Interdisciplinary Technologies, Breast Cancer Research Center, Motamed Cancer Institute, ACECR, Tehran 1125342432, Iran; y.zare@aut.ac.ir; 2 Department of Mechanical Engineering (BK21 Four), College of Engineering, Kyung Hee University, Yongin 17104, Korea; 3 Department of Chemistry, Inha University, Incheon 22212, Korea

**Keywords:** conductive carbon nanotubes (CNTs), polymer nanocomposites, incomplete interface, effective conductivity, interfacial conductivity

## Abstract

Interfacial conductivity and “*L_c_*”, i.e., the least carbon-nanotube (CNT) length required for the operative transfer of CNT conductivity to the insulated medium, were used to establish the most effective CNT concentration and portion of CNTs needed for a network structure in polymer CNT nanocomposites (PCNT). The mentioned parameters and tunneling effect define the effective conductivity of PCNT. The impact of the parameters on the beginning of percolation, the net concentration, and the effective conductivity of PCNT was investigated and the outputs were explained. Moreover, the calculations of the beginning of percolation and the conductivity demonstrate that the experimental results and the developed equations are in acceptable agreement. A small “*L_c_*” and high interfacial conductivity affect the beginning of percolation, the fraction of networked CNTs, and the effective conductivity. Additionally, a low tunneling resistivity, a wide contact diameter, and small tunnels produce a highly effective conductivity. The developed model can be used to optimize breast cancer cell sensors.

## 1. Introduction 

Carbon nanotubes (CNTs) endow significant conductivity and high mechanical properties to composites, especially polymer CNT nanocomposites (PCNT) [1,2,3,4,5,6,7,8,9,10,11,12,13,14,15,16,17,18]. A CNT aspect ratio in a range of 500–1000 creates a conductive network at extremely low concentrations of CNTs [19,20,21,22,23,24,25,26]. Indeed, the beginning of percolation essential for the nanocomposite conductivity. Former studies correlated the percolation threshold with the aspect ratio of nanofiller [23,26], but the construction of CNTs in nanocomposites and the interphase area can govern the beginning of percolation [27,28,29,30]. Recent studies have tried to establish the mentioned parameters in the beginning of percolation of the nanofiller in a PCNT [31,32,33]. In PCNTs, only one phase (CNT) is conductive and so responsible for the conductivity of the whole sample. In fact, the medium in the PCNTs is insulated, simplifying the simulation of nanocomposite conductivity. However, metal matrix nanocomposites containing CNT include two conductive phases. The high concentration of free electrons in the metallic systems causes the electrical conductivity and is, thus, one of the most crucial design criteria for selecting a suitable sample [34]. However, due to the intrinsic complexity of these systems, the accurate determination of electrical conductivity in metal matrix nanocomposites is a significant challenge [34]. 

The tunneling effect is an important factor in nanocomposite conductivity, because neighboring nanoparticles can transfer electrons via the tunneling influence [35,36,37]. Therefore, the tunneling effect does not depend on the linked CNTs, and nanocomposites demonstrate conductivity when CNTs are detached by too low a distance (a maximum of 10 nm). However, this mechanism is rarely considered in the conductivity of systems. Certain modeling studies predicted the conductivity of nanocomposites by estimating the filler, interphase, and tunneling aspects [38,39,40], but many researches used the old equations for the percolation threshold and conductivity. Undoubtedly, the conventional models cannot provide the proper calculations for the conductivity of nanocomposites, due to the main terms being disregarded. Since conductivity plays a crucial role in biosensors based on polymer nanocomposites, a simple model can optimize sensors for breast cancer cells and other sensors.

The mechanical properties of nanocomposites worsen as a result of poor interfacial linkages between the polymer and nanofiller [13,41]. In fact, weak interfacial adhesion impedes any advantages associated with the filler being passed on to the polymer medium. Therefore, the interfacial region in the nanocomposites effectively controls the performance. The interfacial aspects can affect the whole conductivity, because robust interfacial interaction/adhesion allows the conductive filler’s exceptional conductivity to be passed on to the medium, while a weak interface cannot ensure a desirable conductivity is transferred. However, the available studies in this area do not explain the roles of the adhesion at the interface in percolation inception and conductivity. 

In this paper, “*L_c_*” is stated as the minimum length of CNTs needed to provide effective transfer of filler conductivity to the polymer host. Thereafter, “*L_c_*” expresses the effective levels of CNT concentration and length, beginning of percolation, and the proportion of networked CNTs in the nanocomposites. Moreover, the roles of CNT, the interphase, and tunneling parameters in these terms are properly established. In this study, the mentioned parameters and tunneling effect were used to simulate the effective conductivity of PCNT. The impressions of all parameters on the percolation, network fraction, and conductivity terms were investigated using the developed equations and the outputs were justified. The contour plots can help the optimization of parameters to achieve the maximum conductivity in polymer nanocomposites. In addition, a large amount of experimental data were used to approve the calculations. This study is helpful to use the simple and proper equations for the prediction of the beginning of percolation, the net fraction, and effective conductivity in PCNT. All parameters and equations are meaningful and reasonably facilitate the calculations. 

## 2. Theoretical Explanations

An incomplete interfacial linkage in nanocomposites cannot bear high amounts of stress and results in breakages. In this scenario, the high portion of nanotubes is not fully loaded, which weakens their reinforcing effect in nanocomposites [42]. A similar approach can explain the effect of the interface on nanocomposite conductivity. A flawed interface impedes the transfer of the complete filler conductivity (*σ_f_*) to the surrounding medium. Therefore, the polymer–filler interface controls the nanocomposite conductivity. 

“*L_c_*” denotes the smallest length of CNT required to flawlessly transfer the complete conductivity of CNTs to the medium and is defined as follows:

(1) $$L_{c}=\frac{\sigma_{f_{}}D}{2\tau }=\frac{\sigma_{f_{}}R}{\tau }$$

where “*D*” and “*R*” denote the CNT diameter and radius, respectively, and “*τ*” denotes the interfacial conductivity.

When $0\leq x\leq 2L_{c}$, the whole CNT cannot reach the maximum conductivity; however, at $x>2L_{c}$, the whole CNT can reach the maximum conductivity as the entire conductivity is transferred. 

When the CNTs are flawlessly bonded to the polymer medium, the normal conductivity ($\bar{\sigma }$) is equal to “*σ_f_*”, but the imperfect interfacial linkage causes a “$\bar{\sigma }$” that is lower than “*σ_f_*”. Accordingly, the effective CNT length (*l_eff_*) is given by

(2) $$\bar{\sigma }l=\sigma_{f}l_{eff}$$

where “*l*” is CNT length.

At x < 2*L_c_*, “*l_eff_*” and the operative filler amount ($\phi_{eff}$) are defined [42] as follows:

(3) $$l_{eff}=l\frac{l}{4L_{c}}$$

(4) $$\phi_{eff}=\phi_{f}\frac{l}{4L_{c}}$$

where “$\phi_{f}$” is the volume portion of the nanofiller in the sample. 

When *l* > 2*L_c_*, “*l*_eff_” and “$\phi_{eff}$” are expressed [42] as follows:

(5) $$l_{eff}=l(1-\frac{L_{c}}{l})$$

(6) $$\phi_{eff}=\phi_{f}(1-\frac{L_{c}}{l})$$

Assuming two mentioned zones for CNTs at x < 2*L_c_* and x > 2*L_c_*, “*l_eff_*” and “$\phi_{eff}$” are given by

(7) $$l_{eff}=\frac{2L_{c}}{l}(l\frac{l}{4L_{c}})+(\frac{l-2L_{c}}{l})l(1-\frac{L_{c}}{l})=\frac{l}{2}+(l-2L_{c})(1-\frac{L_{c}}{l})$$

(8) $$\phi_{eff}=\frac{2L_{c}}{l}(\phi_{f}\frac{l}{4L_{c}})+(\frac{l-2L_{c}}{l})\phi_{f}(1-\frac{L_{c}}{l})=\phi_{f}[\frac{1}{2}+(\frac{l-2L_{c}}{l^{2}})(l-L_{c})]$$

The effective factors are expressed by the interfacial conductivity via the exchange of “*L_c_*” from Equation (1) into Equations (7) and (8), as follows:

(9) $$l_{eff}=\frac{l}{2}+(l-\frac{2\sigma_{f_{}}R}{\tau })(1-\frac{\sigma_{f_{}}R}{\tau l})$$

(10) $$\phi_{eff}=\phi_{f}[\frac{1}{2}+(\frac{l-\frac{2\sigma_{f_{}}R}{\tau }}{l^{2}})(l-\frac{\sigma_{f_{}}R}{\tau })]$$

However, CNTs experience curliness in the nanocomposites [43], which decreases their effectiveness in the nanocomposites. 

“*l_eq_*” denotes the smallest distance between the two ends of the nanotube and can be written as follows:

(11) $$u=\frac{l}{l_{eq}}$$

where a higher “*u*” than 1 shows increased curliness. 

The curliness also degrades the natural conductivity of CNTs [44]. As a result, the conductivity of curved CNTs is defined by

(12) $$\sigma_{CNT}=\frac{\sigma_{f}}{u}$$

The curliness term from Equations (9) and (10) adjusts the effective issues to

(13) $$l_{eff}=\frac{l}{2u}+(\frac{l}{u}-\frac{2\sigma_{f_{}}R}{u\tau })(1-\frac{\sigma_{f_{}}R}{\tau l})$$

(14) $$\phi_{eff}=\phi_{f}[\frac{1}{2}+(\frac{\frac{l}{u}-\frac{2\sigma_{f_{}}R}{u\tau }}{\frac{l^{2}}{u^{2}}})(\frac{l}{u}-\frac{\sigma_{f_{}}R}{u\tau })]=\phi_{f}[\frac{1}{2}+(\frac{l-\frac{2\sigma_{f_{}}R}{\tau }}{l^{2}})(l-\frac{\sigma_{f_{}}R}{\tau })]$$

The interphase region of the CNTs can widen the networks in the nanocomposites [31,32]. In addition, the interfacial conductivity and curliness modify the CNT’s effective length. Consequently, the beginning of percolation of randomly dispersed CNTs in PCNT is expressed [45] as

(15) $$\phi_{p}=\frac{\pi R^{2}l}{\frac{32}{3}\pi {\left(R+t\right)}^{3}[1+\frac{3}{4}(\frac{l_{eff}}{R+t})+\frac{3}{32}{\left(\frac{l_{eff}}{R+t}\right)}^{2}]}$$

where “*t*” is the interphase thickness. 

Furthermore, only a proportion of CNTs is involved in the network after the beginning of percolation. The portion of CNTs in the network [40] is calculated by

(16) $$f=\frac{\phi_{f}^{1/3}-\phi_{p}^{1/3}}{1-\phi_{p}^{1/3}}$$

When the operative filler portion is substituted into Equation (16), “*f*” is developed as follows:

(17) $$f=\frac{\phi_{eff}^{1/3}-\phi_{p}^{1/3}}{1-\phi_{p}^{1/3}}$$

This can be used to calculate the network volume fraction in the nanocomposites, as follows:

(18) $$\phi_{N}=f\phi_{eff}^{}=(\frac{\phi_{eff}^{1/3}-\phi_{p}^{1/3}}{1-\phi_{p}^{1/3}})\phi_{f}[\frac{1}{2}+(\frac{l-\frac{2\sigma_{f_{}}R}{\tau }}{l^{2}})(l-\frac{\sigma_{f_{}}R}{\tau })]$$

The operative resistance of composites with tip-to-tip platelets is defined [46] as

(19) $$\rho_{eff}=R_{sample}\frac{wh}{L}$$

where “*w*”, “*h*”, and “*L*” denote the width, thickness, and length of the sample, respectively. In addition, “*R_sample_*”, which is the whole resistance of the sample, is defined by

(20) $$R_{sample}=\frac{LTR_{t}}{\phi_{f}wh}$$

where “*T*” is the thickness of platelets. Moreover, “*R_t_*” denotes the nanoparticle and tunneling resistances.

Substituting Equation (20) into Equation (19) results in

(21) $$\rho_{eff}=\frac{TR_{t}}{\phi_{f}}$$

Inverting the previous equation expresses the effective conductivity as

(22) $$\sigma_{eff}=\frac{\phi_{f}}{TR_{t}}$$

which considers the involvement of all particles in the sample conductivity; however, only the network phase improves the conductivity of nanocomposites. 

The volume portion of the networked filler ($\phi_{N}$) and the filler diameter develop Equation (22) into

(23) $$\sigma_{eff}=\frac{\phi_{N}}{2RR_{t}}$$

The total resistance of each path includes the CNT resistance (*R_f_*) and the tunneling resistance (*R_tun_*) as

(24) $$R_{t}=R_{f}+R_{tun}$$

(25) $$R_{f}=\frac{l}{\pi R^{2}\sigma_{f}}$$

Considering Equations (12) and (13) in the previous equation results in

(26) $$R_{f}=\frac{l_{eff}u}{\pi R^{2}\sigma_{f}}$$

In addition, “*R_tun_*” consists of the resistances of CNTs (*R_1_*) and the insulated polymer (*R_2_*) in the tunneling zone as follows:

(27) $$R_{tun}=R_{1}+R_{2}$$

“*R_1_*” is defined [47] by

(28) $$R_{1}=\frac{1}{d\sigma_{f}}$$

where “*d*” denotes the contact diameter between close CNTs. 

The curliness boosts the “*R_1_*” as

(29) $$R_{1}=\frac{u}{d\sigma_{f}}$$

Moreover, “*R_2_*” is defined as

(30) $$R_{2}=\frac{\rho \lambda }{S}≅\frac{\rho \lambda }{d^{2}}$$

“*ρ*” denotes the polymer layer tunneling resistivity, “*λ*” denotes the tunneling length/distance, and “*S*” denotes the contact area. 

Therefore, the previous equations define the “*R_tun_*” as

(31) $$R_{tun}=\frac{u}{d\sigma_{f}}+\frac{\rho \lambda }{d^{2}}$$

When Equations (26) and (31) are substituted into Equation (24), the whole resistance is expressed by

(32) $$R_{t}=\frac{l_{eff}u}{\pi R^{2}\sigma_{f}}+\frac{u}{d\sigma_{f}}+\frac{\rho \lambda }{d^{2}}$$

Substituting “$\phi_{N}$” and “*R_t_*” from Equations (18) and (32) into Equation (23) creates a model for the effective conductivity, as follows:

(33) $$\sigma_{eff}=\frac{\left(\frac{\phi_{eff}^{1/3}\;-\;\phi_{p}^{1/3}}{1\;-\;\phi_{p}^{1/3}})\phi_{f}[\frac{1}{2}+(\frac{l\;-\;\frac{2\sigma_{f_{}}R}{\tau }}{l^{2}})(l-\frac{\sigma_{f_{}}R}{\tau })\right]}{2R(\frac{l_{eff}u}{\pi R^{2}\sigma_{f}}+\frac{u}{d\sigma_{f}}+\frac{\rho \lambda }{d^{2}})}$$

This equation establishes the components of the CNT network, the interface conductivity, the interphase depth, and the tunneling properties in the effective conductivity of PCNT. In the present modeling, the interphase is considered as a separate phase for the conductivity of samples. This model is accurate because the size and conductivity of all the effective components, such as the interphase, CNT, and tunneling zone, are considered. 

## 3. Results and Discussion

In this section, advanced equations are used to explain the stimuli of various factors on the beginning of percolation, the network proportion, and the effective conductivity. The average values of the factors in this study are considered as $\phi_{f}$ = 0.02, *u* = 1.2, *σ_f_* = 10^5^ S/m, *l* = 15 μm, *R* = 10 nm, *t* = 10 nm, *τ* = 400 S/m, *d* = 20 nm, *λ* = 2 nm, and *ρ* = 100 Ω∙m. 

### 3.1. Percolation Threshold 

**Figure 1:**
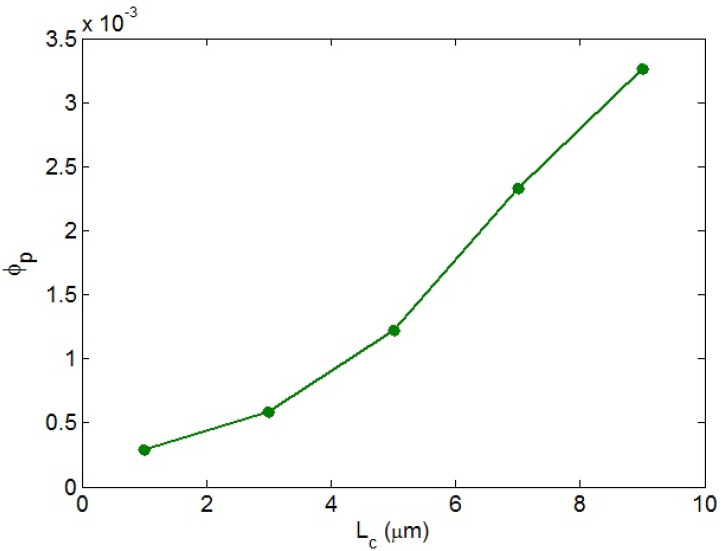
The beginning of percolation of CNTs in PCNT as “*L_c_*” increases.

Figure 1 demonstrates the role of “*L_c_*” in the beginning of percolation of nanoparticles. It is obvious that the percolation threshold increased as “*L_c_*” grows. In fact, a desirable beginning of percolation was observed at a low “*L_c_*”, but a high “*L_c_*” increased the percolating level. *L_c_* = 1 μm resulted in a percolation threshed of approximately 0.0003, while *L_c_* = 9 μm produced a percolation threshold of 0.0033. As a result, only a low level of “*L_c_*” caused a small percolation threshold. A low “*L_c_*” indicated that the maximum conductivity of CNTs was easily transferred to the surrounding medium. In other words, a low “*L_c_*” demonstrated that the bonds between the polymer host and incorporated CNTs were robust and the conductivity had been transferred perfectly. In this scenario, the CNT’s effective length (Equation (13)) increased, causing a low percolation threshold. In fact, a low “*L_c_*”, demonstrating a perfect interfacial adhesion, produced large effective CNTs, which diminishes the percolation threshold. However, a high “*L_c_*”, demonstrating poor interfacial bonding, worsened the effective CNT length, which increased the percolation inception. Therefore, the developed equation demonstrates the role of “*L_c_*” in the beginning of percolation. 

**Figure 2:**
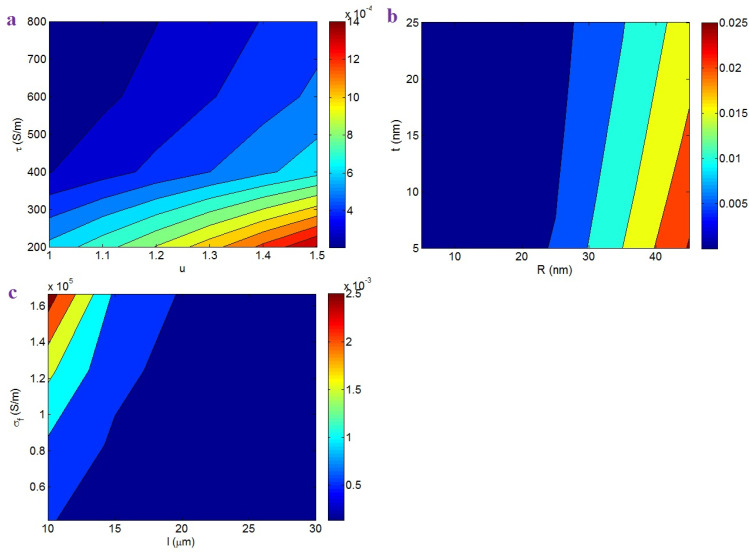
Expression of the beginning of percolation (Equation (15)) at different levels of (**a**) “*u*” and “*τ*”, (**b**) “*R*” and “*t*”, and (**c**) “*l*” and “*σ_f_*” parameters by contour plots.

The contour plots also illustrate the impacts of various factors on the beginning of percolation. Figure 2a shows the beginning of percolation at different “*u*” and “*τ*” values. The maximum percolation level of 0.00014 is shown at *u* = 1.5 and *τ* = 200 S/m, while *u* < 1.14 and *τ* > 600 S/m caused the lowest percolation threshold of 0.0002. Accordingly, the percolation threshold was lower at lower curliness and a higher interfacial conductivity. Instead, a higher curliness and a poorer interfacial conductivity seriously increased the beginning of percolation. A high curliness reduced the effective CNT length in the nanocomposites and, therefore, the curliness undesirably affected the extent of percolation. However, a poorer curliness shows that the CNTs in nanocomposites were straighter, which created networks from small number of nanoparticles. Moreover, a high range of interfacial attachment increased the effective length of CNTs (Equation (13)). Since large CNTs can form conductive networks at a low CNT concentration, a high interfacial conductivity inhibited the beginning of percolation. On the basis of these descriptions, the advanced equations meaningfully show the roles of “*u*” and “*τ*” in the percolation level. 

Figure 2b depicts the effects of “*R*” and “*t*” on the beginning of percolation. The highest percolation level of 0.025 is detected at *R* = 45 nm and *t* = 5 nm, nevertheless the percolation threshold significantly reduced at *R* < 27 nm. Hence, thin CNTs and a thick interphase yielded a poor percolation threshold in the samples. These outputs are logical, because thin CNTs induced a low “*L_c_*”, which facilitated the formation of conductive networks. In fact, thin CNTs caused a large effective CNT length in the nanocomposites, which definitely manipulated the beginning of percolation. However, thick CNTs decreased the effective length, which considerably increased percolation. Additionally, a dense interphase formed the large interphase region contributing to the networks in the nanocomposites. Indeed, a dense interphase around CNTs efficiently linked the nanoparticles at a low filler concentration. Consequently, the roles of “*R*” and “*t*” in the beginning of percolation are logical.

Figure 2c demonstrates the impact of CNT length and conductivity on the beginning of percolation. A low percolation threshold is obtained by long CNTs (high *l*), whereas the beginning of percolation significantly increased as a result of short CNTs and a high CNT conductivity. Therefore, long CNTs were sufficient to achieve a low percolation threshold, while short CNTs and high filler conductivity increased it. It is clear that percolation and networking in bigger CNTs were easier to achieve than in shorter ones, because the distance between CNTs decreased as their length increases. That is to say that short CNTs cannot percolate at a low concentration and a large number of CNTs is required to build the networks. As a result, large CNTs formed conductive networks at low concentrations. However, high filler conductivity increased the percolation threshold, because a large “*L_c_*” was obtained by a high “*σ_f_*”. In fact, the high CNT conductivity enhanced the “*L_c_*”, decreasing the operational CNT length. Therefore, high filler conductivity resulted in an undesirable percolation threshold. It is obvious that large CNTs dominantly affected the percolation threshold (Equation (15)), because their role in the effective length was more important than that of filler conductivity (see Equation (13)). 

### 3.2. Percentage of Networked CNTs

**Figure 3:**
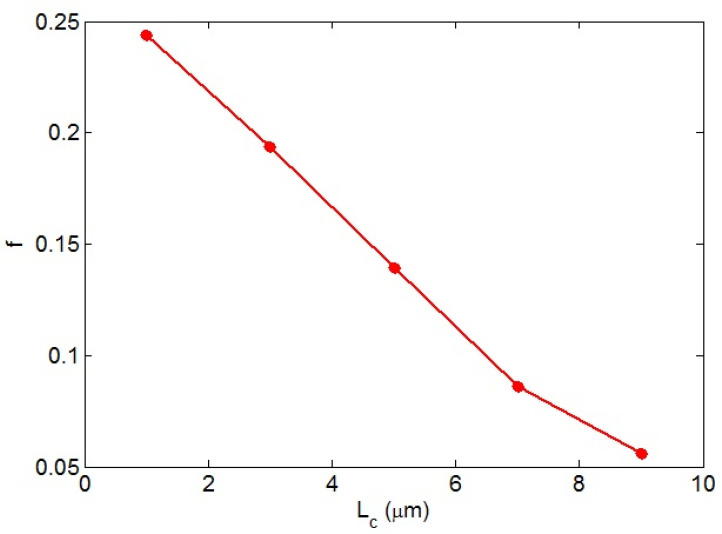
“*f*” levels at different values of “*L_c_*” based on Equation (17).

In this section, the impacts of various parameters on the CNT network percentage after the percolation threshold (*f*) is met are explained. Figure 3 displays the variation of “*f*” at different “*L_c_*” values based on Equation (17). A low “*L_c_*” caused a high “*f*”, but “*f*” reduced considerably at a high “*L_c_*”. Accordingly, high levels of “*L_c_*” negatively affected the networked CNTs in the PCNT. A high “*L_c_*” increased the percolation threshold, as described in the previous section. Moreover, a large “*L_c_*” produced poor bonds between the medium and nanoparticles, which reduced the operative CNT concentration (Equation (14)). Accordingly, a high “*L_c_*”, which increased the beginning of percolation and a low effective filler concentration, logically reduced the fraction of networks in PCNT. 

**Figure 4:**
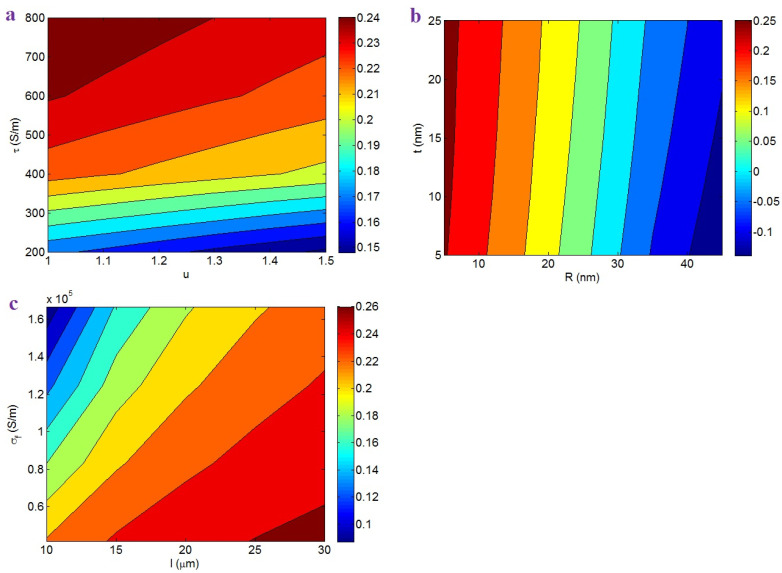
“*f*” depending on (**a**) “*u*” and “*τ*”, (**b**) “*R*” and “*t*”, and (**c**) “*l*” and “*σ_f_*” factors.

Figure 4a shows the “*f*” levels at different amounts of “*u*” and “*τ*”. A low “*u*” and high “*τ*” produce a high “*f*”, while a high “*u*” and low “*τ*” decrease the value of “*f*”. Therefore, poor curvature and strong interfacial conductivity expanded the CNT networks. More curliness and poorer interfacial conductivity produced a smaller fraction of networked CNTs. The curliness did not affect the effective filler concentration (Equation (14)), but a low curliness significantly reduced the beginning of percolation. Moreover, a higher interfacial conductivity induced a poorer percolation threshold (see Figure 2a). Additionally, a higher interfacial conductivity produced a large effective filler amount (Equation (14)). On the basis of Equation (17), a higher percentage of networked CNTs was achieved by a more effective CNT concentration and poorer percolation. Accordingly, low curliness and good interfacial conductivity produced a high “*f*”, as defined by Equation (17). 

Figure 4b exemplifies the “*f*” by “*R*” and “*t*”. The maximum *f* = 0.25 is obtained at *R* = 5 nm, whereas *R* = 45 nm and *t* < 18 nm primarily decreased the “*f*”. Therefore, thin CNTs produced a high proportion of CNTs in the networks, but thick CNTs and a thin interphase mostly reduced it. Thin CNTs decreased the percolation threshold in PCNT, as observed in the previous section. Moreover, thin CNTs caused a high effective filler concentration, because the narrow CNTs reduced the “*L_c_*”. In reality, thin CNTs can increase the filler efficiency in the composites. Therefore, the role of thin CNTs in “*f*” is logical. Moreover, a big interphase decreased the beginning of percolation (see Figure 2b). Since a poor beginning of percolation produced large networks in the samples, the dependency of “*f*” on “*t*” was correctly predicted by the developed equation.

Figure 4c also reveals the importance of CNT length and natural conductivity on the percentage of CNTs in the network. Large CNTs and poor filler conductivity produced large networks, but short CNTs and high CNT conductivity produced small ones. As a result, big networks were produced by large CNTs and low filler conductivity. Large CNTs significantly reduced the beginning of percolation and increased the effective filler content. Therefore, long CNTs were able to produce big networks in the samples. Furthermore, low filler conductivity can govern the percolation level and the effective concentration of CNTs, because it reduces the “*L_c_*” (Equation (1)). In other words, a poorer filler conductivity induced a poorer beginning of percolation and increased the operative filler content, which expanded the networks in the nanocomposites. Consequently, the developed equations explain the roles of “*l*” and “*σ_f_*” in the percentage of networked CNTs. 

### 3.3. Effective Conductivity

**Figure 5:**
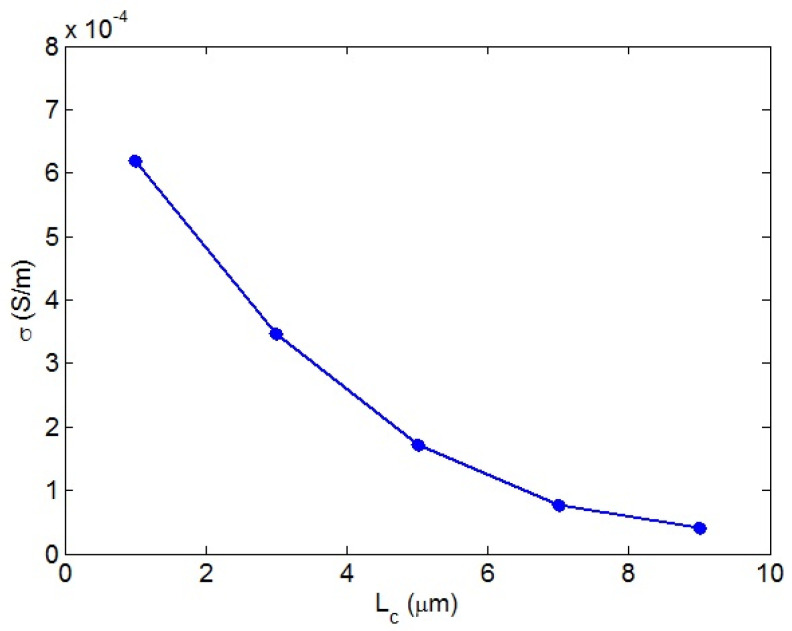
Effective conductivity at different ranges of “*L_c_*”.

The effective conductivity of PCNT at different values of all parameters is expressed in this section according to Equation (33). Figure 5 displays the effect of “*L_c_*” on the effective conductivity. It was found that the effective conductivity declined at a high “*L_c_*”. In other words, a large “*L_c_*” negatively affected the effective conductivity. A high “*L_c_*” produced a poor interface/interphase, which means that the conduction was poorly transferred due to the poor interfacial connections among the polymer chains and nanoparticles [45,48]. In fact, the poor interface failed to efficiently transport the excellent CNT conductivity to the polymer medium, which weakened the effective conductivity. A high level of “*L_c_*” negatively affected the beginning of percolation and the network fraction, which worsened the effective conductivity. Therefore, the “*L_c_*” value significantly affects the interfacial conductivity and the effective conductivity, and the lowest “*L_c_*” produces the highest conductivity. According to these explanations, the correlation between effective conductivity and “*L_c_*” is as expected. 

**Figure 6:**
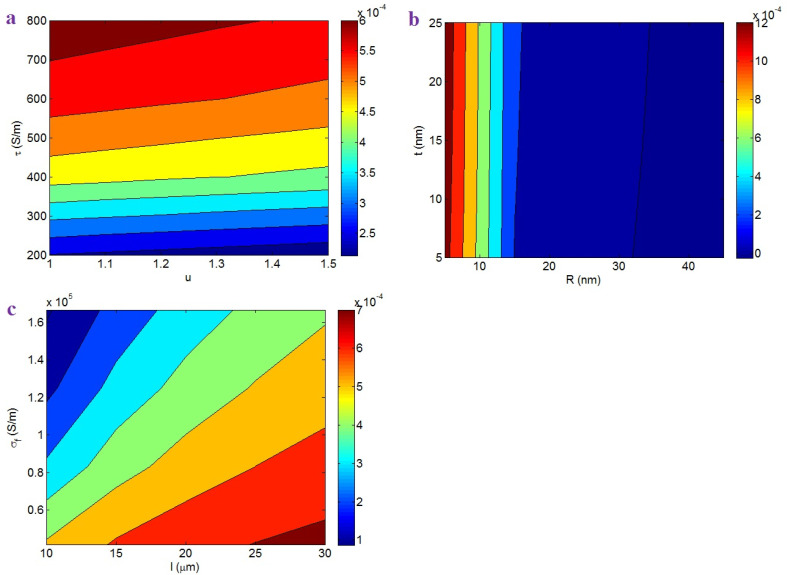
Dependencies of nanocomposite effective conductivity (Equation (33)) on (**a**) “*u*” and “*τ*”, (**b**) “*R*” and “*t*”, and (**c**) “*l*” and “*σ_f_*” factors.

Figure 6a exhibits the roles of “*u*” and “*τ*” in the effective conductivity. The most effective conductivity was attained at the smallest “*u*” and the highest “*τ*”. As a result, the effective conductivity improved as a result of low curliness and high interface conductivity. Conversely, a high curliness and a slight conductivity of interface largely weakened the effective conductivity. These results are reasonable due to the impact of CNT curliness and interface conductivity on the operative terms. The poor curliness intensified the effective values of CNT length and concentration in the nanocomposites. Therefore, it is reasonable to expect a higher effective conductivity at lower CNT curliness. In addition, the high conductivity of interface effectively transferred the CNT conduction to the surrounding medium, which improved the effective conductivity. In fact, high interfacial conductivity produced a highly effective filler concentration and big networks, which enhanced the effective conductivity. Accordingly, the proposed equation correctly predicts the impacts of “*u*” and “*τ*” on the effective conductivity.

Figure 6b depicts the outputs of the effective conductivity at different ranges of “*R*” and “*t*”. “*R*” inversely affected the effective conductivity, but “*t*” did not play a significant role. Therefore, thinner CNTs produced a more effective conductivity; however, the interphase depth did not change the effective conductivity. A small CNT radius positively affected the operative CNT length and concentration, the percolation threshold, and the size of the networks. In other words, thin CNTs created big and dense networks in PCNT. As a result, the correlation between the effective conductivity and CNT radius is meaningful. However, a dense interphase changed the beginning of percolation, but a very low beginning of percolation insignificantly affected the “*f*”. Accordingly, the interphase thickness cannot control the effective conductivity. 

Figure 6c shows the effective conductivity at different values of “*l*” and “*σ_f_*” using Equation (33). The most effective conductivity was obtained at the highest “*l*” and the poorest “*σ_f_*”, while the smallest “*l*” and the highest “*σ_f_*” reduced the effective conductivity. Therefore, long CNTs and a poor filler conductivity provided a desirable effective conductivity in PCNT. The long CNTs decreased the beginning of percolation and produced big networks. Accordingly, it was expected that the large CNTs would increase the effective conductivity. Moreover, a high CNT conductivity increased the “*L_c_*”, which negatively affected the beginning of percolation, the operative filler concentration, and the network size. In fact, the CNTs hardly transferred the high conductivity to the polymer medium when a poor interface was present; therefore, effective conductivity was not developed. In summary, the advanced model correctly predicts the effects of the mentioned factors on the effective conductivity. 

**Figure 7:**
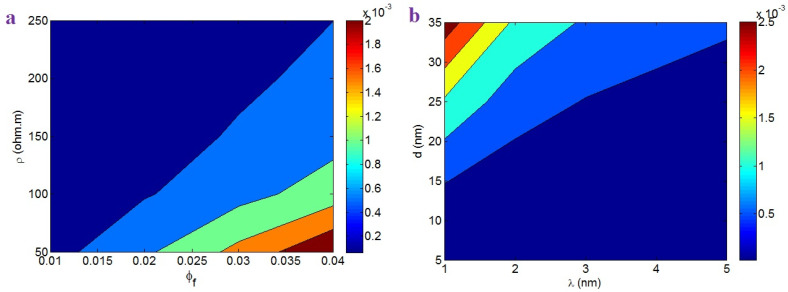
Variations of effective conductivity according to the (**a**) “$\phi_{f}$” and “*ρ*”; and (**b**) “λ” and “*d*” factors.

Figure 7a shows the outputs of Equation (33) at different series of “$\phi_{f}$” and “*ρ*”. This plot indicates that a high “$\phi_{f}$” and a poor “*ρ*” increased the effective nanocomposite conductivity, while a small “$\phi_{f}$” and high “*ρ*” produced a poor effective conductivity. Therefore, the filler amount and tunneling resistivity directly and inversely affected the effective conductivity, respectively. A higher CNT amount yielded a higher effective conductivity, because the large number of CNTs produced big and dense conductive networks. Moreover, the tunneling resistivity directly affected the tunneling resistance (Equation (31)), i.e., a high tunneling resistivity enhanced the tunneling resistance, which significantly reduced charge transportation via the tunneling region. Since the effective conductivity mainly depends on the tunneling properties, an undesirable high tunnel resistivity in the effective conductivity is functional. 

Figure 7b depicts the impacts of “*λ*” (tunneling distance) and “*d*” (contact diameter) on the effective conductivity. A higher effective conductivity was achieved with a shorter tunneling distance and larger contact diameter. In addition, a large tunneling distance and small contact width considerably reduced the effective conductivity. These outputs were also expected, as small and large tunnels decrease the tunneling resistance and promote charge transfer. However, large-diameter and low-diameter tunnels weaken charge transfer, as the distant CNTs and the low contact area reduce the ability to transport electrons. Hence, the developed model logically predicts the role of the tunneling properties in the effective conductivity. 

### 3.4. Comparison between Predictions and Experimental Data

The predictions of the developed equations were compared with the experimental measurements of the percolation threshold and conductivity for several samples from the literature. The experimental data of four samples including poly (vinyl chloride) (PVC)/multi walled CNT (MWCNT) (*R* = 8 nm, *l* = 16 μm, *u* = 1.2) [49], poly (ethylene terephthalate) (PET)/MWCNT (*R* = 5 nm, *l* = 1 μm, *u* = 1.2) [50], epoxy/single walled CNT (SWCNT) (*R* = 1 nm, *l* = 2 μm, *u* = 1.6) [51], and polycarbonate (PC)/acrylonitrile butadiene styrene (ABS)/MWCNT (*R* = 5 nm, *l* = 1.5 μm, *u* = 1.2) [52] are considered. “$\phi_{p}$” was reported as 0.0005, 0.001, 0.0003, and 0.002 for the PVC/MWCNT, PET/MWCNT, epoxy/SWCNT, and PC/ABS/MWCNT samples, respectively. These levels were compared with the predictions of Equation (15). The values of (*t*, *τ*) were obtained as (1.5, 400), (3, 300), (7, 145), and (8, 1600) (nm, S/m) for the PVC/MWCNT, PET/MWCNT, epoxy/SWCNT, and PC/ABS/MWCNT samples, respectively. These results are meaningful, which validates the results from the proposed equation for the percolation threshold, assuming the interfacial conductivity and interphase thickness. The thickest interphase was observed for the PC/ABS/MWCNT nanocomposite, but the PVC/MWCNT nanocomposite produced the smallest “*t*”. The varying properties of the interphase regions demonstrate the formation of different interfaces/interphases in the reported samples. The values of “*L_c_*” were also obtained using Equation (1) as 1.67, 1.39, 0.43, and 0.26 μm for the PVC/MWCNT, PET/MWCNT, epoxy/SWCNT, and PC/ABS/MWCNT samples, respectively. The lowest “*L_c_*” was observed in the PC/ABS/MWCNT sample, but the PVC/MWCNT nanocomposite produced the largest “*L_c_*”. These results indicate that the CNT conductivity was effectively transferred to the polymer matrix in the PC/ABS/MWCNT sample, while poor conductivity transport was revealed in the PVC/MWCNT nanocomposite.

**Figure 8:**
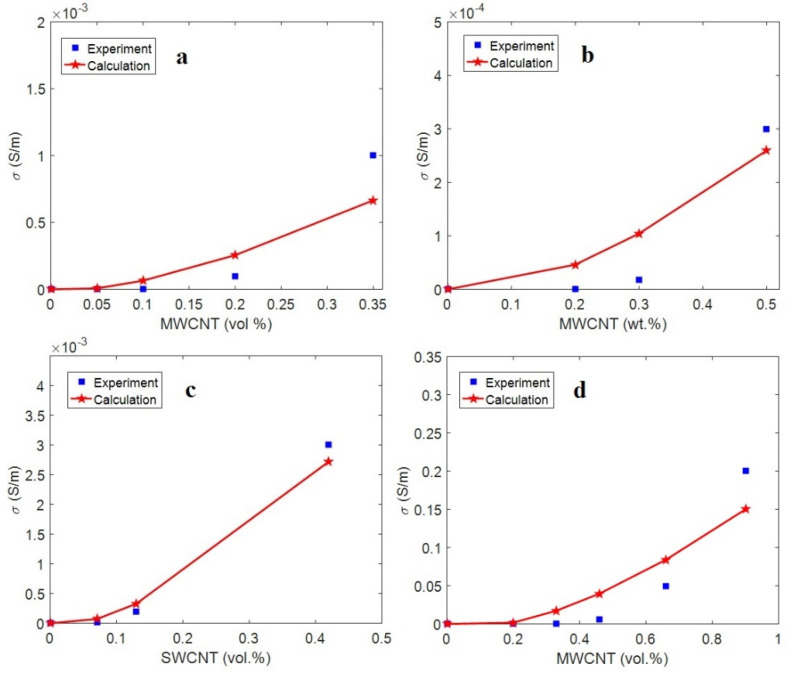
Experimental data and calculations of the effective conductivity using the developed model for the (**a**) PVC/MWCNT [49], (**b**) PET/MWCNT [50], (**c**) epoxy/SWCNT [51], and (**d**) PC/ABS/MWCNT [52] samples.

The above calculations were used in Equation (33) to predict the effective conductivity of the reported samples. Figure 8 shows the experimental results and the predictions of the effective conductivity for the samples. The calculations of the effective conductivity demonstrate a good agreement with the experimental results, which confirms the developed model. The contact diameter was calculated as 22, 18, 20, and 35 nm for the PVC/MWCNT, PET/MWCNT, epoxy/SWCNT, and PC/ABS/MWCNT samples, respectively. Moreover, the tunneling distance was calculated as 2, 3, 2, and 0.5 nm for the PVC/MWCNT, PET/MWCNT, epoxy/SWCNT, and PC/ABS/MWCNT samples, respectively. In addition, “*ρ*”, which is the polymer layer tunneling resistivity, was calculated as 8, 30, 8, and 1 Ω∙m for the PVC/MWCNT, PET/MWCNT, epoxy/SWCNT, and PC/ABS/MWCNT samples, respectively. All these levels are meaningful, which validates the efficacy of the proposed equations. These data indicate the highest tunneling resistance in the PET/MWCNT sample (Equation (31)). In contrast, the PC/ABS/MWCNT nanocomposite produced the minimum tunneling resistance. The lowest conductivity of the PET/MWCNT sample and the highest conductivity of the PC/ABS/MWCNT nanocomposite show that the tunneling zone plays a central role in the effective conductivity of nanocomposites.

## 4. Conclusions

The beginning of percolation, the portion of networked filler, and the effective conductivity were expressed based on “*L_c_*” and interfacial conductivity. Furthermore, the advanced equations were utilized to describe the impacts of all factors related to the CNTs, the interphase, and the tunneling region on the mentioned terms. In addition, the calculations of conductivity demonstrated a good agreement with the experimental results, validating the results from the developed model. A low “*L_c_*”, a low curliness, a high interface conductivity, a poor CNT conductivity, thin and long CNTs, and a dense interphase caused a low beginning of percolation and a high effective filler amount. In fact, these levels produced big networks in PCNT. Therefore, these optimized levels improved the effective conductivity of PCNT. The importance of parameters on the declared terms was assessed, confirming the validity of the equations. In addition, a poor polymer layer tunneling resistivity, short tunnels, and a big contact diameter produced a highly effective conductivity, as they reduced the tunneling resistance, facilitating charge transport. The developed model can be applied to optimize breast cancer cell sensors, since electrical conductivity plays a central role in biosensors containing polymer nanocomposites.

## Data Availability

The data presented in this study are available on request from the corresponding author.
